# Association of Ankle-Brachial Index and Aortic Arch Calcification with Overall and Cardiovascular Mortality in Hemodialysis

**DOI:** 10.1038/srep33164

**Published:** 2016-09-09

**Authors:** Szu-Chia Chen, Mei-Yueh Lee, Jiun-Chi Huang, Ming-Chen Paul Shih, Jer-Ming Chang, Hung-Chun Chen

**Affiliations:** 1 Division of Nephrology, Department of Internal Medicine, Kaohsiung Medical University Hospital, Kaohsiung Medical University, Kaohsiung, Taiwan; 2 Department of Internal Medicine, Kaohsiung Municipal Hsiao-Kang Hospital, Kaohsiung Medical University, Kaohsiung, Taiwan; 3 Faculty of Medicine, College of Medicine, Kaohsiung Medical University, Kaohsiung, Taiwan; 4 Division of Endocrinology and Metabolism, Department of Internal Medicine, Kaohsiung Medical University Hospital, Kaohsiung Medical University, Kaohsiung, Taiwan; 5 Department of Medical Imaging, Kaohsiung Medical University Hospital, Kaohsiung Medical University, Kaohsiung, Taiwan; 6 Department of Medical Imaging, Kaohsiung Medical University Hospital, Kaohsiung, Taiwan Department of Radiology, Faculty of Medicine, Taiwan

## Abstract

Peripheral artery occlusive disease and vascular calcification are highly prevalent in hemodialysis (HD) patients, however the association of the combination of ankle-brachial index (ABI) and aortic arch calcification (AoAC) with clinical outcomes in patients undergoing HD is unknown. In this study, we investigated whether the combination of ABI and AoAC is independently associated with overall and cardiovascular mortality in HD patients. The median follow-up period was 5.7 years. Calcification of the aortic arch was assessed by chest X-ray. Forty-seven patients died including 24 due to cardiovascular causes during the follow-up period. The study patients were stratified into four groups according to an ABI < 0.95 or ≥0.95 and an AoAC score of >4 or ≤4 according to receiver operating characteristic curve. Those with an ABI < 0.95 and AoAC > 4 (*vs.* ABI ≥ 0.95 and AoAC score ≤ 4) were associated with overall (hazard ratio [HR], 4.913; 95% confidence interval [CI], 1.932 to 12.497; *p* = 0.001) and cardiovascular (HR, 3.531; 95% CI, 1.070 to 11.652; *p* = 0.038) mortality in multivariable analysis. The combination of a low ABI and increased AoAC was associated with increased overall and cardiovascular mortality in patients undergoing HD.

Peripheral artery occlusive disease (PAOD) is highly prevalent in hemodialysis (HD) patients^1^,^2^, and the ankle-brachial index (ABI) is a simple and noninvasive method that can be used to identify PAOD. A low ABI has been reported to be a reliable diagnostic marker for PAOD with high sensitivity and specificity, and also to be a strong predictor for overall and cardiovascular mortality in HD patients^3^,^4^,^5^. We also previously reported an association between a low ABI and vascular access failure^6^. Both traditional risk factors such as age and diabetes and nontraditional risk factors such as hypocalcaemia and hyperphosphatemia have been associated with a higher risk of amputation, suggesting that vascular calcification contributes to PAOD in this population^7^,^8^.

Vascular calcification is very common in patients with end-stage renal disease, and especially in those undergoing HD^9^. Vascular calcification can be assessed using several imaging modalities including computed tomography (CT), ultrasonography and plain X-rays^10^,^11^. HD patients often undergo chest radiography because it offers a quick estimation of the dimensions of the heart, and also information on calcification of the aortic arch. Aortic arch calcification (AoAC) on a chest X-ray may represent total AoAC, and it has been associated with cardiovascular and all-cause mortality among patients with end-stage renal disease^12^,^13^. Previous studies have confirmed the relationship between vascular calcification and atherosclerosis in chronic renal failure patients^14^,^15^. Adragao^14^ and colleagues found that vascular calcification in aorta and iliac-femoral axis were associated with an ABI < 0.9 14. Besides, An *et al*.^15^ showed chronic renal failure patients with a high vascular calcification score had more atherosclerotic calcified plaques in the femoral or popliteal artery. However, no previous studies have evaluated the association between the combination of ABI and AoAC and clinical outcomes in patients undergoing HD. Therefore, the aim of this study was to assess whether the combination of ABI and AoAC is independently associated with overall and cardiovascular mortality in patients undergoing HD.

## Study Patients and Methods

### Study Patients and Design

This study enrolled all patients receiving a maintenance HD program, thrice weekly over 3 months, in a dialysis clinic of a regional hospital in Taiwan in August 2010. The exclusion criteria were as follows: (1) patients who refused any ABI-form device examination (n = 5), (2) patients with atrial fibrillation (n = 4), (3) patients with bilateral below-knee amputation (n = 2), (4) patients without chest X-rays (n = 17), and (5) patients with hospitalization or antibiotic treatment in the last 4 weeks (n = 5) were excluded. In total, 197 patients (90 males and 107 females; mean age 58.0 ± 12.5 years) were included as the study group. All of the patients received HD three times per week, with each HD session lasting for 3.5–4.5 hours with a blood flow rate of 250–300 mL/min and dialysate flow of 500 mL/min. Blood samples were taken before and after HD to calculate Kt/V. The study protocol was approved by the Institutional Review Board of Kaohsiung Medical University Hospital, and all participants provided written informed consent to participate in this study. The methods were carried out in accordance with the approved guidelines.

### Assessment of ABI

ABI was measured 10–30 minutes before HD using an ABI-form device which automatically and simultaneously measured blood pressure in both arms and ankles using an oscillometric method^16^. Occlusion and monitoring cuffs were placed tightly around the upper arm without blood access and both sides of the lower extremities in the supine position. ABI was calculated as the ratio of the systolic blood pressure at the ankles divided by the systolic blood pressure at the arms with the lowest value of ankle systolic blood pressure being used for the calculation. The ABI was measured in August 2010.

### Evaluation of AoAC and Cardiothoracic Ratio (CTR) by Chest X-Ray

One experienced radiologist blinded to the clinical data of the patients reviewed the chest plain films of the HD patients. Calcification of the aortic arch was assessed using a scale developed by Ogawa *et al*.^17^, with the aortic arch divided into 16 sections by circumference on chest X-rays, and the number of sections with calcification being counted. The CTR was defined as the ratio of a transverse diameter of the cardiac shadow to the transverse diameter of the chest on chest X-ray, with cardiomegaly defined as CTR > 50%. Chest X-rays were performed in August 2010.

### Collection of Demographic, Medical and Laboratory Data

Demographic and medical data including age, gender, and comorbid conditions were obtained from medical records and interviews with the patients. Body mass index was calculated as the ratio of weight in kilograms divided by the square of the height in meters. Laboratory data were measured from fasting blood samples using an AutoAnalyzer (Roche Diagnostics GmbH, D-68298 Mannheim COBAS Integra 400). Blood samples were obtained within 1 month of enrollment. Kt/V was evaluated monthly as a marker of dialysis efficiency and determined according to the Daugirdas procedure^18^.

### Definition of Overall and Cardiovascular Mortality

Overall and cardiovascular deaths were confirmed and ascertained from medical records by two cardiologists, with disagreements resolved by a third cardiologist. Patients were followed until death, and the remaining patients were followed until February 2016.

### Reproducibility

Thirty patients were randomly selected to evaluate the reproducibility of AoAC by one experienced radiologist and one trained medical doctor. The mean percent error was calculated as the absolute difference divided by the average of the two observations.

### Statistical analysis

Statistical analysis was performed using SPSS version 17.0 for Windows (SPSS Inc., Chicago, USA). Data were expressed as percentages, mean ± standard deviation, or median (25^th^–75^th^ percentile) for the duration of dialysis, and levels of triglycerides and intact parathyroid hormone. We used receiver operating characteristic (ROC) curve to get the cut-off value of ABI and AoAC for outcomes. Multiple comparisons among the study groups were performed using one-way analysis of variance followed by a Bonferroni-adjusted post hoc test. Associations between the study groups and overall and cardiovascular mortality were assessed using multivariable Cox proportional hazard analysis. Significant variables in the univariable analysis were selected for multivariable analysis. Patients with an ABI ≥ 0.95 and AoAC score ≤ 4 were defined as the reference group, and had the lowest risk of mortality. Survival curves for overall and cardiovascular mortality were obtained using Kaplan-Meier analysis. A difference was considered to be statistically significant at *p* < 0.05.

## Results

Of the 197 included patients, we stratified the patients into four groups according to an ABI < 0.95 or ≥0.95 and a median AoAC score of >4 or ≤4 according to ROC curve. The mean percent error for AoAC measurements was 12.3 ± 12.3%. A comparison of the clinical characteristics of these groups is shown in Table 1. Compared to the patients with an ABI ≥ 0.95 and AoAC score ≤ 4, those with an ABI < 0.95 and AoAC score > 4 were older, had higher prevalence of diabetes, coronary artery disease and cerebrovascular disease, high prevalence of CTR > 50%, lower levels of albumin and creatinine and higher levels of total calcium.

Compared to the patients with an AoAC score ≤ 4, those with an AoAC score > 4 had a lower ABI (0.91 ± 0.21 *vs.* 1.00 ± 0.17, *p* = 0.002) and higher prevalence of ABI < 0.95 (53.2% *vs.* 28.8%, *p* = 0.001).

### Risk of Overall Mortality

Overall, the median follow-up period was 5.7 years. During the follow-up period, 47 patients died (23.9%), including 24 due to cardiovascular causes, four due to malignancy, 10 due to infectious diseases, two due to gastrointestinal bleeding, and seven due to other causes. Table 2 lists the hazard ratios (HRs) of variables for overall mortality. The univariable regression analysis shows that the group with an ABI < 0.95 and AoAC score > 4 (*vs.* the group with an ABI ≥ 0.95 and AoAC score ≤ 4; HR, 6.865; 95% confidence interval [CI], 3.169 to 14.871; *p* < 0.001), old age, a history of diabetes, coronary artery disease and cerebrovascular disease, low albumin, low total cholesterol, and low creatinine are associated with a significant increase in overall mortality. In the multivariable analysis, the group with an ABI < 0.95 and AoAC score > 4 (*vs.* the group with an ABI ≥ 0.95 and AoAC score ≤ 4; HR, 4.913; 95% CI, 1.932 to 12.497; *p* = 0.001), and low albumin (per 0.1 g/dL; HR, 0.786; 95% CI, 0.683 to 0.904; *p* = 0.001) are associated with overall mortality.

**Figure 1:**
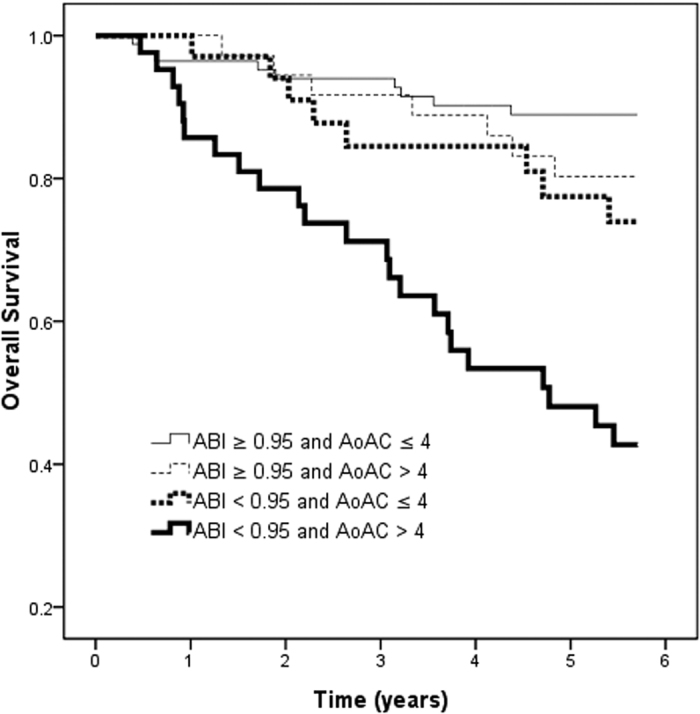
Kaplan-Meier analyses of overall survival (log-rank *p* < 0.001) among 4 study groups. The group with ABI < 0.95 and AoAC score > 4 had a worse overall survival than that with ABI ≥ 0.95 and AoAC score ≤ 4.

Figure 1 illustrates the Kaplan-Meier survival curves for overall survival among the four study groups. The patients with an ABI < 0.95 and AoAC score > 4 had worse overall survival than with an ABI ≥ 0.95 and AoAC score ≤ 4 (log-rank test, *p* < 0.001).

We have further used ABI < 0.95 and AoAC > 4 as separate variables in the multivariable forward analysis, and find that ABI < 0.95 (HR, 3.206; 95% CI, 1.662 to 6.184; *p* = 0.001), and AoAC > 4 (per 0.1 g/dL; HR, 1.975; 95% CI, 1.037 to 3.764; *p* = 0.029) are associated with increased overall mortality.

### Risk of Cardiovascular Mortality

Of those who died due to cardiovascular causes during the follow-up period, 11 died due to heart failure, two due to myocardial infarction, six due to fatal arrhythmia, and five due to hemorrhagic stroke. Cox proportional hazards regression analysis of the four study groups for cardiovascular mortality is shown in Table 3. The univariable regression analysis shows that the group with an ABI < 0.95 and AoAC score > 4 (*vs.* the group with an ABI ≥ 0.95 and AoAC score ≤ 4; HR, 5.898; 95% CI, 2.041 to 17.041; *p* = 0.001), old age, a history of diabetes and coronary artery disease, low albumin, low total cholesterol, and low creatinine are associated with a significant increase in cardiovascular mortality. In the multivariable analysis, the group with an ABI < 0.95 and AoAC score > 4 (*vs.* the group with an ABI ≥ 0.95 and AoAC score ≤ 4; HR, 3.531; 95% CI, 1.070 to 11.652; *p* = 0.038), and low albumin (per 0.1 g/dL; HR, 0.813; 95% CI, 0.676 to 0.977; *p* = 0.027) are associated with cardiovascular mortality.

**Figure 2:**
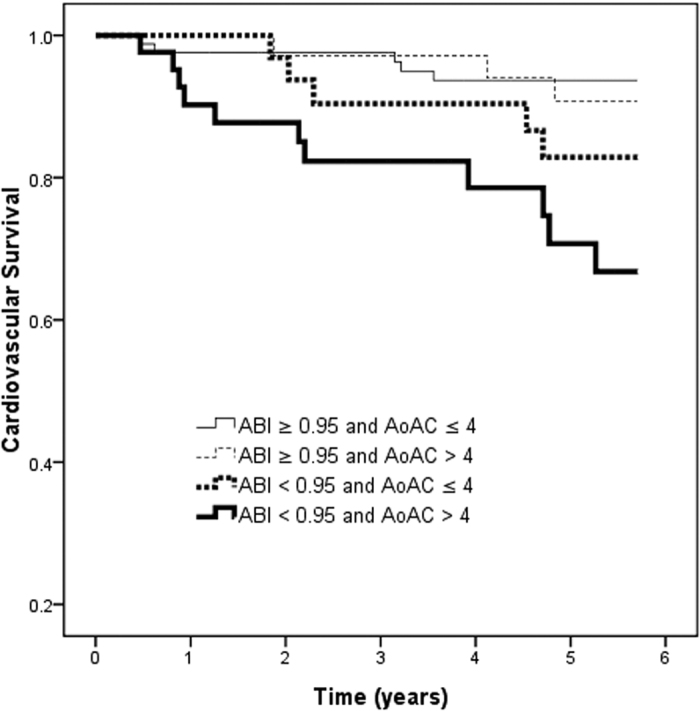
Kaplan-Meier analyses of cardiovascular survival (log-rank *p* = 0.001) among 4 study groups. The group with ABI < 0.95 and AoAC score > 4 had a worse cardiovascular survival than that with ABI ≥ 0.95 and AoAC score ≤ 4.

Figure 2 shows the Kaplan-Meier survival curves for cardiovascular survival among the four study groups. The patients with an ABI < 0.95 and AoAC score > 4 had a worse cardiovascular survival than with an ABI ≥ 0.95 and AoAC score ≤ 4 (log-rank test, *p* = 0.001).

We have further used ABI < 0.95 and AoAC > 4 as separate variables in the multivariable forward analysis, and find that ABI < 0.95 (HR, 4.092; 95% CI, 1.661 to 10.083; *p* = 0.002) is associated with increased cardiovascular mortality, but AoAC > 4 is not.

To further identify whether there is the interaction effect between ABI and AoAC, we perform analysis to show the relation of ABI < 0.95 to overall and cardiovascular mortality under AoAC > 4 and AoAC ≤ 4 in Table 4. In the multivariable analysis, the group with an ABI < 0.95 and AoAC score > 4 is associated with increased overall (HR, 3.694; 95% CI, 1.412 to 9.668; *p* = 0.008) and cardiovascular mortality (HR, 6.194; 95% CI, 1.241 to 30.923; *p* = 0.026), but not in the group with an ABI < 0.95 and AoAC score ≤ 4.

## Discussion

In this study we evaluated the association between a combination of ABI and AoAC and clinical outcomes in patients undergoing HD. The results showed that the patients with an ABI < 0.95 and AoAC score > 4 were associated with increases in overall and cardiovascular mortality compared to those with an ABI ≥ 0.95 and AoAC score ≤ 4.

The known risk factors for vascular calcification are increasing age, dialysis, hypertension, diabetes, dyslipidemia, inflammation, malnutrition, oxidative stress, hyperphosphatemia and elevated calcium-phosphate product^19^,^20^,^21^, all of which are also risk factors for atherosclerosis^22^. An *et al*.^15^ compared the association between vascular calcification score on plain radiographs of the feet and atherosclerotic calcified plaques of the femoral or popliteal artery with Doppler ultrasonography in pre-dialysis, HD and peritoneal dialysis patients. They found that the patients with a high vascular calcification score had more atherosclerotic calcified plaques in the femoral or popliteal artery. Besides peripheral artery, Adragao *et al*.^14^ evaluated the association between ABI and abdominal aorta calcification score using plain abdominal X-rays in HD patients, and found that the vascular calcification score in central arteries was associated with an ABI < 0.9. In the present study, we also found that HD patients with a higher AoAC score measured from plain chest X-rays had a lower ABI and higher prevalence of ABI < 0.95. The association between AoAC score from plain X-rays with ABI emphasizes the relationship between AoAC and the extension of atherosclerosis in peripheral arteries.

An important finding in the present study is that a combination of low ABI and increased AoAC was significantly associated with increased overall and cardiovascular mortality in patients undergoing HD. The precise mechanisms responsible for the relationship between the combination of AoAC and atherosclerosis and mortality are not completely understood, although involvement of structural and functional changes within the heart have been proposed. PAOD, vascular calcification and left ventricular hypertrophy (LVH) are highly prevalent in HD patients^2^,^9^,^21^. In addition, many factors including pressure overload, renal anemia, malnutrition, and inflammatory status have been reported to be both main causes of the development of LVH and also for atherosclerosis in patients undergoing HD^23^,^24^. Furthermore, previous studies have demonstrated significantly lower ABI values in patients with LVH than in those without LVH, and that ABI was independently and inversely associated with left ventricular mass index^25^,^26^. These results suggest that a low ABI may be related to LVH. Atherosclerosis has also been reported to directly cause a decrease in blood perfusion in the lower extremities and an increase in arterial wall stiffness, contributing to a lower ABI and arterial distensibility, and then ultimately LVH^1^,^27^,^28^. In contrast, LVH has been reported to cause left ventricular systolic and diastolic dysfunction and a decrease in cardiac output, which further worsens deficiencies in blood perfusion of the extremities and promotes the progression of peripheral arterial disease resulting in a decrease in ABI. In addition, vascular calcification induces arterial wall stiffness and reduces vascular compliance, which in turn is correlated with increased left ventricular afterload and hypertrophy^29^. Coronary artery calcium score, aorta calcium score, and AoAC volume as measured on CT have been reported to be independently associated with arterial stiffening, LVH, and left ventricular diastolic dysfunction, respectively^10^,^30^. Hence, LVH and left ventricular diastolic dysfunction may represent a causal intermediary between AoAC and atherosclerosis with adverse outcomes.

In this study, compared to the patients with an ABI ≥ 0.95 and AoAC score ≤ 4, those with an ABI < 0.95 and AoAC score > 4 had a higher prevalence of risk factors for cardiovascular morbidity, including older age, higher prevalence of diabetes, coronary artery disease and cerebrovascular disease and high prevalence of CTR >. Even after adjusting for these confounding factors, the patients with an ABI < 0.95 and AoAC score > 4 were still associated with an increase in overall and cardiovascular mortality. Hence, we can identify a group of HD patients at high-risk of adverse outcomes using low ABI and increased AoAC.

We have also performed analysis using ABI < 0.95 and AoAC > 4 as separate variables into multivariable analysis, and find the similar findings. ABI < 0.95 is still associated with increased overall and cardiovascular mortality in the multivariable analysis. AoAC > 4 is also related to overall mortality, but not cardiovascular mortality in the multivariable analysis. The sample size is an important issue in the statistical analysis. Because there are just 47 deaths and 24 cardiovascular deaths in this study, and there are too many variables in the multivariable analysis. We choose significant variables in univariable analysis, and then put into multivariable analysis. The reason for the non-significant association between AoAC > 4 and cardiovascular mortality may be related to small sample size. The statistical power may be reduced due to the small sample size.

Malnutrition may worsen patient outcome by accelerating atherosclerosis and aggravating inflammation^31^. Low serum albumin level, low body mass index, and hypocholesterolemia have been regarded as malnutrition status. Our study showed that low serum albumin level was significantly associated with increased overall and cardiovascular mortality, which was consistent with this previous observation.

There are several limitations to this study. First, the causal relationship between ABI and AoAC could not be confirmed due to the cross-sectional design. Prospective studies are warranted to address this issue. Second, while cardiac CT can accurately and quantitatively evaluate the extent of cardiovascular calcification, it is costly and involves exposure to radiation. AoAC score is a semi-quantitative tool used to evaluate AoAC on chest X-rays. It is a simple and non-invasive tool that has been shown to be highly correlated with AoAC volume by multi-detector CT^18^. However, although plain radiography is highly correlated with the AoAC volume as determined by multi-detector CT^18^, cardiac CT can accurately and quantitatively evaluate the extent of cardiovascular calcification than plain radiograph. Third, plain radiography is also not sensitive enough to detect early-stage vascular calcification. Fourth, the limited number of study patients severely reduced the power of the study. In addition, evaluating AoAC using a semi-quantitative method is relatively crude. Finally, as no studies have documented the reliable abnormal values of AoAC, we used ROC curve to get the cut-off value of AoAC to classify our study patients.

In conclusion, our results demonstrate that the combination of a low ABI and high AoAC was associated with increased overall and cardiovascular mortality in patients undergoing HD. ABI and AoAC assessments might be useful in identifying a group of HD patients at high-risk of adverse outcomes.

## Additional Information

**How to cite this article**: Chen, S.-C. *et al*. Association of Ankle-Brachial Index and Aortic Arch Calcification with Overall and Cardiovascular Mortality in Hemodialysis. *Sci. Rep.* **6**, 33164; doi: 10.1038/srep33164 (2016).

## Figures and Tables

**Table 1:** Comparison of clinical characteristics among study groups.

Characteristics	ABI ≥ 0.95 and AoAC ≤ 4 (n = 84)	ABI ≥ 0.95 and AoAC > 4 (n = 37)	ABI < 0.95 and AoAC ≤ 4 (n = 34)	ABI < 0.95 and AoAC > 4 (n = 42)
Age tertile (year)				
<53	48.8	24.3*	23.5*,†	7.1*,†,#
53–63	35.7	35.1	38.2	23.8
≥63	15.5	40.5	38.2	69.0
Male gender (%)	47.6	43.2	58.8	33.3
Duration of dialysis (years)	4.2 (2.5–9.0)	8.9 (3.8–11.0)*	5.9 (2.6–7.5)	6.5 (3.8–10.6)
Diabetes mellitus (%)	26.2	40.5	58.8*	52.4*
Hypertension (%)	69.0	72.3	70.0	78.3
Coronary artery disease (%)	9.5	24.3	35.3*	33.3*
Cerebrovascular disease (%)	1.2	5.4	20.6*	19.0*
Systolic blood pressure (mmHg)	146.8 ± 28.0	153.0 ± 27.4	156.9 ± 26.3	151.6 ± 29.5
Diastolic blood pressure (mmHg)	82.3 ± 16.1	81.0 ± 14.9	83.9 ± 16.0	74.8 ± 15.6
ABI	1.08 ± 0.09	1.08 ± 0.09	0.79 ± 0.15*,†	0.75 ± 0.16*,†
CTR > 50% (%)	48.8	64.9	50.0	78.6*
Body mass index (kg/m^2^)	23.8 ± 3.6	23.6 ± 3.4	25.8 ± 3.8*	23.9 ± 3.9
Laboratory parameters				
Albumin (g/dL)	4.0 ± 0.3	3.9 ± 0.3	3.9 ± 0.2	3.8 ± 0.3*
Triglyceride (mg/dL)	113.5 (79.8–186.8)	115.5 (89.3–177.8)	168 (112.8–260)	137 (97–168)
Total cholesterol (mg/dL)	176.8 ± 41.7	173.4 ± 47.9	187.1 ± 38.9	179.9 ± 40.6
HDL-cholesterol (mg/dL)	41.6 ± 11.8	40.0 ± 10.9	38.7 ± 11.3	40.1 ± 9.8
LDL-cholesterol (mg/dL)	81.0 ± 28.8	84.7 ± 31.6	93.9 ± 28.4	87.5 ± 25.5
Hemoglobin (g/dL)	10.1 ± 1.4	9.9 ± 1.5	10.3 ± 1.2	10.2 ± 1.4
Creatinine (mg/dL)	10.9 ± 2.3	9.9 ± 2.2	10.7 ± 2.3	9.0 ± 2.2*,#
Total calcium (mg/dL)	9.0 ± 0.9	9.1 ± 0.9	9.2 ± 0.6	9.6 ± 1.0*
Phosphorous (mg/dL)	4.8 ± 1.2	4.2 ± 1.3	4.5 ± 1.3	4.7 ± 1.2
Calcium-phosphorous product (mg^2^/dL^2^)	42.9 ± 10.4	38.1 ± 12.7	41.4 ± 12.8	44.8 ± 13.2
Uric acid (mg/dL)	7.5 ± 1.5	7.3 ± 1.8	7.9 ± 1.3	7.1 ± 1.7
iPTH (pg/mL)	294 (143.9–504)	285.4 (151.6–604.9)	330.7 (197.3–473.2)	373.4 (155.4–627.4)
Kt/V	1.6 ± 0.3	1.6 ± 0.3	1.5 ± 0.3	1.6 ± 0.3

Abbreviations. ABI, ankle-brachial index; AoAC, aortic arch calcification; CTR, cardiothoracic ratio; HDL, high-density lipoprotein; LDL, low-density lipoprotein; iPTH, intact parathyroid hormone.

The study patients were stratified into 4 groups according to ABI < 0.95 or ≥ 0.95 and median score of AoAC (3).

*p < 0.05 compared ABI ≥ 0.95 and AoAC ≤ 4; ^†^*p* < 0.05 compared with ABI ≥ 0.95 and AoAC > 4; ^#^*p* < 0.05 compared with ABI < 0.95 and AoAC ≤ 4.

**Table 2:** Relation of study groups to overall mortality using Cox proportional hazards model.

Parameters	Univariable		Multivariable	
	HR (95% CI)	*p*	HR (95% CI)	*p*
Study groups				
ABI ≥ 0.95 and AoAC ≤ 4	1		1	
ABI ≥ 0.95 and AoAC > 4	1.774 (0.661–4.763)	0.255	1.487 (0.497–4.453)	0.478
ABI < 0.95 and AoAC ≤ 4	2.417 (0.932–6.267)	0.069	2.314 (0.828–6.462)	0.110
ABI < 0.95 and AoAC > 4	6.865 (3.169–14.871)	<0.001	4.913 (1.932–12.497)	0.001
Age tertile (year)				
<53	1		1	
53–63	2.765 (0.996–7.677)	0.051	2.124 (0.721–6.259)	0.172
≥63	6.142 (2.368–15.932)	<0.001	2.396 (0.778–7.381)	0.128
Male gender	1.310 (0.739–2.321)	0.355	—	—
Duration of dialysis (per 1 year)	0.980 (0.486–1.975)	0.955	—	—
Diabetes mellitus	2.337 (1.310–4.169)	0.004	1.494 (0.783–2.852)	0.223
Hypertension	1.602 (0.775–3.314)	0.204	—	—
Coronary artery disease	1.970 (1.066–3.639)	0.030	1.095 (0.541–2.214)	0.801
Cerebrovascular disease	3.017 (1.456–6.251)	0.003	1.075 (0.462–2.499)	0.867
Systolic blood pressure (per 1 mmHg)	1.005 (0.995–1.015)	0.304	—	—
Diastolic blood pressure (per 1mmHg)	0.989 (0.971–1.008)	0.266	—	—
CTR > 50%	1.711 (0.926–3.161)	0.086	—	—
Body mass index (per 1 kg/m^2^)	0.975 (0.901–1.055)	0.530	—	—
Laboratory parameters				
Albumin (per 0.1 g/dL)	0.785 (0.714–0.862)	<0.001	0.786 (0.683–0.904)	0.001
Triglyceride (per 1 mg/dL)	0.999 (0.996–1.002)	0.438	—	—
Total cholesterol (per 1 mg/dL)	0.991 (0.983–0.999)	0.024	0.995 (0.986–1.003)	0.189
HDL-cholesterol (per 1 mg/dL)	0.986 (0.959–1.014)	0.331	—	—
LDL-cholesterol (per 1 mg/dL)	0.996 (0.986–1.006)	0.467	—	—
Hemoglobin (per 1 g/dL)	0.900 (0.720–1.125)	0.356	—	—
Creatinine (per 1 mg/dL)	0.800 (0.698–0.917)	0.001	1.135 (0.950–1.356)	0.163
Total calcium (per 1 mg/dL)	1.124 (0.805–1.569)	0.493	—	—
Phosphorous (per 1 mg/dL)	0.984 (0.775–1.250)	0.894	—	—
Calcium-phosphorous product (per 1 mg^2^/dL^2^)	1.001 (0.977–1.026)	0.928	—	—
iPTH (per 1 pg/mL)	1.367 (0.695–2.690)	0.365	—	—
Uric acid (per 1 mg/dL)	0.856 (0.699–1.048)	0.132	—	—
Kt/V (per 1)	0.368 (0.116–1.172)	0.091	—	—

Values express as hazard ratios (HR) and 95% confidence interval (CI). Abbreviations are same as Table 1.

**Table 3:** Relation of study groups to cardiovascular mortality using Cox proportional hazards model.

Parameters	Univariable		Multivariable	
	HR (95% CI)	*p*	HR (95% CI)	*p*
Study groups				
ABI ≥ 0.95 and AoAC ≤ 4	1		1	
ABI ≥ 0.95 and AoAC > 4	1.369 (0.327–5.728)	0.667	0.725 (0.137–3.837)	0.705
ABI < 0.95 and AoAC ≤ 4	2.713 (0.785–9.378)	0.115	2.495 (0.686–9.071)	0.165
ABI < 0.95 and AoAC > 4	5.898 (2.041–17.041)	0.001	3.531 (1.070–11.652)	0.038
Age tertile (year)				
<53	1		1	
53–63	4.448 (0.961–20.589)	0.056	3.333 (0.694–16.015)	0.133
≥63	7.180 (1.617–31.892)	0.010	2.195 (0.427–11.289)	0.347
Male gender	1.257 (0.565–2.798)	0.576	—	—
Duration of dialysis (per 1 year)	1.291 (0.460–3.627)	0.628	—	—
Diabetes mellitus	2.862 (1.252–6.543)	0.013	1.335 (0.542–3.288)	0.529
Hypertension	1.882 (0.643–5.506)	0.248	—	—
Coronary artery disease	2.502 (1.094–5.723)	0.030	1.343 (0.536–3.366)	0.529
Cerebrovascular disease	2.499 (0.852–7.327)	0.095	—	—
Systolic blood pressure (per 1 mmHg)	1.007 (0.993–1.021)	0.313	—	—
Diastolic blood pressure (per 1mmHg)	0.998 (0.974–1.024)	0.896	—	—
CTR > 50%	1.945 (0.806–4.692)	0.139	—	—
Body mass index (per 1 kg/m^2^)	0.995 (0.891–1.110)	0.924	—	—
Laboratory parameters				
Albumin (per 0.1 g/dL)	0.772 (0.679–0.877)	<0.001	0.813 (0.676–0.977)	0.027
Triglyceride (per 1 mg/dL)	0.995 (0.989–1.001)	0.128	—	—
Total cholesterol (per 1 mg/dL)	0.985 (0.974–0.997)	0.012	0.988 (0.974–1.001)	0.068
HDL-cholesterol (per 1 mg/dL)	0.983 (0.945–1.023)	0.395	—	—
LDL-cholesterol (per 1 mg/dL)	0.992 (0.977–1.006)	0.247	—	—
Hemoglobin (per 1 g/dL)	0.807 (0.587–1.108)	0.184	—	—
Creatinine (per 1 mg/dL)	0.820 (0.679–0.989)	0.038	—	—
Total calcium (per 1 mg/dL)	0.918 (0.592–1.424)	0.703	—	—
Phosphorous (per 1 mg/dL)	1.106 (0.797–1.535)	0.547	—	—
Calcium-phosphorous product (per 1 mg^2^/dL^2^)	1.009 (0.976–1.044)	0.586	—	—
iPTH (per 1 pg/mL)	1.983 (0.727–5.408)	0.181	—	—
Uric acid (per 1 mg/dL)	0.928 (0.702–1.228)	0.603	—	—
Kt/V (per 1)	0.204 (0.039–1.056)	0.058	—	—

Values express as hazard ratios (HR) and 95% confidence interval (CI). Abbreviations are same as Table 1.

**Table 4:** Relation of ABI < 0.95 to overall and cardiovascular mortality under AoAC > 4 and AoAC ≤ 4.

Parameters	Univariable		Multivariable		*p* for interaction
	HR (95% CI)	*p*	HR (95% CI)	*p*	
Overall mortality					
AoAC > 4	3.945 (1.688–9.218)	0.002	3.694 (1.412–9.668)	0.008	0.608
AoAC ≤ 4	2.379 (0.917–6.171)	0.075	1.775 (0.527–5.973)	0.354	
Cardiovascular mortality					
AoAC > 4	4.405 (1.223–15.868)	0.023	6.194 (1.241–30.923)	0.026	0.507
AoAC ≤ 4	2.696 (0.779–9.323)	0.117	2.673 (0.699–10.228)	0.151	

Values express as hazard ratios (HR) and 95% confidence interval (CI). Abbreviations are same as Table 1.
